# Folate Deficiency Is Spatially Dependent and Associated with Local Farming Systems among Women in Ethiopia

**DOI:** 10.1093/cdn/nzac088

**Published:** 2022-05-03

**Authors:** Binyam G Sisay, Hasset Tamirat, Fanny Sandalinas, Edward J M Joy, Dilenesaw Zerfu, Adamu Belay, Liberty Mlambo, Murray Lark, E Louise Ander, Dawd Gashu

**Affiliations:** Center for Food Science and Nutrition, Addis Ababa University, Addis Ababa, Ethiopia; Center for Food Science and Nutrition, Addis Ababa University, Addis Ababa, Ethiopia; Faculty of Epidemiology and Population Health, London School of Hygiene & Tropical Medicine, London, United Kingdom; Faculty of Epidemiology and Population Health, London School of Hygiene & Tropical Medicine, London, United Kingdom; Food Science and Nutrition Research Directorate, Ethiopian Public Health Institute, Addis Ababa, Ethiopia; Center for Food Science and Nutrition, Addis Ababa University, Addis Ababa, Ethiopia; Food Science and Nutrition Research Directorate, Ethiopian Public Health Institute, Addis Ababa, Ethiopia; School of Biosciences, University of Nottingham, Loughborough, United Kingdom; School of Biosciences, University of Nottingham, Loughborough, United Kingdom; School of Biosciences, University of Nottingham, Loughborough, United Kingdom; Inorganic Geochemistry, Centre for Environmental Geochemistry, British Geological Survey, Nottingham, United Kingdom; Center for Food Science and Nutrition, Addis Ababa University, Addis Ababa, Ethiopia

**Keywords:** Ethiopia, folate, farming system, micronutrients, spatial distribution, women of reproductive age

## Abstract

**Background:**

Folate is essential for the synthesis and integrity of DNA, normal cell formation, and body growth. Folate deficiency among women of reproductive age (WRA) increases the risk of poor birth outcomes including neural tube defect (NTD)-affected pregnancies. Folate status is largely dependent on dietary intakes.

**Objectives:**

We aimed to explore the spatial distribution of biomarkers of folate status and their association with farming systems among nonpregnant WRA in Ethiopia.

**Methods:**

Serum and RBC folate concentration data were derived from the Ethiopia National Micronutrient Survey of 2015. The spatial dependencies of folate concentration of WRA were investigated and its relation with the dominant local farming system was explored.

**Results:**

The median serum folate and RBC folate concentrations were 12.3 nmol/L and 567.3 nmol/L, respectively. The national prevalence of folate deficiency using homocysteine concentration as a metabolic indicator based on serum and RBC folate concentration was 11.6% and 5.7%, respectively. The majority of women (77.9%) had low RBC folate concentrations consistent with increased risk of NTD-affected pregnancies. Folate nutrition was spatially dependent at distances of ≤ 300 km. A marked variability in folate concentration was observed between farming systems: greater RBC folate concentration (median: 1036 nmol/L) was found among women from the Lake Tana fish-based system, whereas the lowest RBC folate concentration (median: 386.7 nmol/L) was observed in the highland sorghum chat mixed system.

**Conclusions:**

The majority (78%) of WRA in Ethiopia had low folate status potentially increasing the risk of NTD-affected pregnancies. These findings may help national and subnational nutrition intervention strategies to target the most affected areas in the country.

## Teaser

Folate deficiency is highly prevalent among women in Ethiopia. This study also reports spatial dependencies of the folate concentration of women and its relation with the dominant local farming system.

## Introduction

It is estimated that > 2 billion people globally are affected by the deficiency of ≥ 1 micronutrients. Unlike protein energy malnutrition, the consequences of micronutrient deficiency, also known as “hidden hunger,” are typically not visible but manifest as impaired physical and mental health, weakened immune systems and exacerbated infections, and decreased productivity. Micronutrient deficiency affects people of all age groups but children and women are the most vulnerable. The nutritional status of a woman not only influences her health but also is determinant of fetal growth and development, with subsequent impacts throughout the child's life-course (1).

Folate (vitamin B-9) is a water-soluble vitamin important for optimal health and development in humans. It is a cofactor of several enzymes involved in the methylation of biomolecules such as lipids, amino acids, and DNA. It participates in key neurodevelopment processes (2). Folate deficiency has been linked with incidence of chronic diseases such as cardiovascular disease, cancer, and the progression of cognitive impairment in older people (3, 4). Reports also indicate an association between periconceptional folate deficiency and risk of congenital disabilities, especially neural tube defects (NTDs) (5). Folate deficiency can also lead to megaloblastic anemia in adults and children through impaired DNA synthesis and cell division, leading to ineffective erythropoiesis (6). Maternal anemia and NTD-affected pregnancies are among the most important health outcomes of folate deficiency. Maternal anemia is highly prevalent in Ethiopia (7) but reports specific to anemia due to folate deficiency are not available. Also, there are scarce national data on NTDs in Ethiopia. However, available small-scale studies suggest the presence of a high rate of NTDs. For example, a cross-sectional hospital-based study reported the presence of 126 NTDs/10,000 births (8). In addition, a pooled analysis of 15 studies in Ethiopia found 63.3 NTD cases (spinal bifida, anencephaly, and encephalocele) per 10,000 births (9). Both studies reported that folic acid supplementation during the first trimester of pregnancy was associated with protection against NTDs. Another study reported an even higher burden of NTDs (131/10,000 births) in the Tigray region of Ethiopia (10).

For comparison, there were an estimated 260,100 NTD-affected birth outcomes worldwide, i.e., 18.6 cases/10,000 live births, in 2015 (11). Rates vary by country and region, with the following estimated median NTD prevalence rates per 10,000 births between 1990 and 2014: 11.7 for Africa, 21.9 for the Eastern Mediterranean, 9.0 for Europe, 11.5 for the Americas, 15.8 for South-East Asia, and 6.9 for Western Pacific (12). Notably, NTD surveillance systems are typically inadequate in low-income countries, which likely leads to underestimated prevalence (12). A high prevalence of NTDs was also reported in northern China between 2000 and 2004 (120/10,000 births), which was significantly reduced (31.5/10,000 births) in 2014, in response to a folic acid supplementation program (13).

Folate nutritional status among the high-risk groups, women of reproductive age (WRA) and young children, is receiving attention because it is associated with adverse birth and developmental effects, and public health measures are warranted. The WHO recommends daily supplementation of folic acid for all women planning to conceive, continuing until 12 wk into the pregnancy. In addition, many countries mandate mass fortification of wheat flour with folic acid, but this is not the case in Ethiopia where fortification is voluntary (14). Furthermore, the WHO recommends that pregnant women take daily multimicronutrient supplements including folic acid to improve maternal and perinatal health outcomes (15).

There are limited data on folate status of WRA in many parts of the world. The available evidence indicates that folate deficiency is a major nutritional problem among WRA. In several low- and middle-income countries > 20% of WRA have folate deficiency. However, the prevalence of folate deficiency was < 5% in high-income countries (16). It is difficult to determine the extent of folate deficiencies across Africa among WRA owing to scarcity of evidence. However, the available evidence shows that folate deficiency is prevalent among women in Africa and its prevalence varies widely depending on the region (17, 18), from as high as 86.1% in Côte d'Ivoire (19) to < 1% in Democratic Republic of the Congo (20). A study in the Ethiopian population during 2005 that assessed the folate status of WRA (*n* = 970) from 9 out of 11 regions reported the presence of severe deficiency (serum folate concentration < 10 nmol/L) and marginal deficiency (serum folate = 10–15 nmol/L) in 46.1% and 21.2% of the population, respectively (21). Another study, in northwest Ethiopia, assessed serum folate concentrations of school-age children, finding a deficiency prevalence of 14% (22). On the other hand, only 2% of women in their late pregnancy from southern Ethiopia had low plasma folate (23).

Previous studies have indicated spatial variability of mineral micronutrients in humans (24–26), influenced by soil physico-chemical characteristics and environmental variables (27). Unlike mineral micronutrients that are absorbed from the soil, plants synthesize folate de novo. To this end, there is great interest in breeding crops with enhanced folate synthesis to increase dietary folate intakes (28). In low-income settings, many households rely on subsistence and local food production. This indicates the importance of local farming and food systems in delivering adequate nutrient supplies to communities. A recent study in Ethiopia reported that the dominant type of farming system in the area where individuals reside contributed 48.2%, 57.2%, and 26.7% of the intercluster variations in the usual total dietary intakes of vitamin A, iron, and zinc, respectively (29). Estimates of the folate dietary supply indicate the prevalence of deficiency across sub-Saharan Africa was estimated as > 20% in 2005 (30).

Ethiopia is characterized by diverse landscapes and agro-ecological conditions and 16 distinct farming systems have been described within the country (31). Understanding the relation between the micronutrient status of populations and the farming systems on which they depend may help inform food system interventions to increase the sustainable supply of nutrients. The present study aims to assess the magnitude of folate deficiency among WRA in Ethiopia and explore its association with farming systems.

## Methods

### Study design and sample population

**FIGURE 1:**
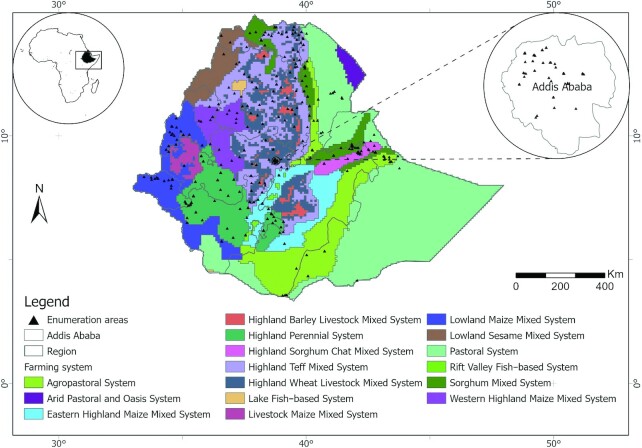
Locations of Ethiopia National Micronutrient Survey enumeration areas (*n* = 346) overlaid on dominant farming systems. Farming system data were obtained from the Famine Early Warning System Network (FEWS NET; https://fews.net) and IFPRI Harvest Choice (https://harvestchoice.org/products/data) databases.

The Ethiopian National Micronutrient Survey (ENMS) was a population-based cross-sectional survey conducted during 2015. The sampling covered all the regions and administrative cities in the country. The survey included young children (6–59 mo old, *n* = 1100), school-age children (5–15 y old, *n* = 1500), WRA (15–49 y old, *n* = 1670), and adult men (15–54 y old, *n* = 500) from enumeration areas as defined by the Central Statistical Agency for the Ethiopian 2007 population and housing census (32) (**Figure 1**). Detailed descriptions of the study design and sampling procedure of the ENMS have been provided elsewhere (24, 25, 33). The present study is based on the analysis of serum folate (*n* = 1648) and RBC folate (*n* = 1647) concentrations among WRA. In the human body, serum or plasma folate is a representation of recent usual intake, whereas RBC folate is a reflection of folate storage over up to ∼4 mo (34).

The sample design described here allows unbiased estimation of parameters for the population and subpopulation, based on the design. In this study we also report a secondary analysis of the data to map the spatial variation of folate status. This was done using model-based rather than design-based statistical methods (35). The original design gives us robustness in that we know the observations are free of bias. However, in place of a design-based analysis which reflects the design frame, the model-based analysis proceeds on the assumption that the observations are a realization of a spatially dependent Gaussian random field (after data transformation if necessary). This accounts for spatial dependence in the observations, and allows us to produce spatial predictions which are least-squares optimal. 

### Data collection and analysis

#### Sociodemographic and dietary data

Sociodemographic characteristics of participating households were collected using a structured questionnaire. The enumerators and supervisors were trained on data collection and interview technique. The detailed method has been presented elsewhere (24, 25, 33). In the present study, only data of nonpregnant (self-reported) women in the ENMS were considered for analysis.

#### Blood collection, processing, and analysis

Blood was collected following WHO blood sampling guidelines (36). Antecubital venous blood was drawn by trained phlebotomists using vacutainers with clot activator needles. Blood samples were centrifuged at room temperature at 3000 rotations per minute for 10 minutes in the field and serum was separated and transferred into vials. In addition, erythrocyte folate samples were prepared by diluting fresh EDTA whole blood with ascorbic acid solution. The detailed blood collection and processing procedure has been described elsewhere (37).

Serum and RBC folate concentrations were assessed using a microbiological assay by which the detection was based on turbidimetric bacterial growth of *Lactobacillus rhamnosus* incubated at 37°C for 42 h (38, 39). For quality control (QC), QC specimens prepared and processed at CDC Atlanta from pooled serum and whole-blood hemolysate, representing low, medium, and high folate concentrations (25, 40, and 60 nmol/L for serum folate; and 300, 400, and 600 nmol/L for whole-blood folate), were used. The limit of detection (LOD) for serum and whole-blood folate was measured as 3 × SD of 10 operational blanks and the limit of quantification (LOQ) was calculated as 10 × SD. The LOD and LOQ were 3.72 and 12.41 nmol/L, respectively. Accuracy was verified by the use of 3 (low, medium, and high folate concentration) pooled serum and whole-blood folate QC specimens. These were prepared in the same way as the samples and typically run at each batch assay. Average recovery for serum folate (*n* = 11) when compared to QC values determined was 98.6%, 99.1%, and 100.1% for low, medium, and high folate concentration, respectively. The recovery rate for whole-blood folate was 102.8%, 105.6%, and 104.3%, for low, medium, and high folate concentration, respectively. The samples and the QCs were analyzed in quintuplicate and if their CV was > 15%,  the samples were repeated. Serum folate is an indicator of acute folate status, whereas RBC folate concentration is a measure of longer-term folate nutrition status (40). Folate deficiency was defined as serum folate < 10 nmol/L and RBC folate < 340 nmol/L using homocysteine concentrations as a metabolic indicator (34). This is because a low concentration of folate is not able to provide a sufficient amount of its methyl group to convert homocysteine to methionine, hence an increase in homocysteine concentration is used as an indicator of folate deficiency. Furthermore, RBC folate insufficiency was determined according to the WHO guidelines cutoff for the optimal RBC folate concentration for NTD risk reductions (<906 nmol/L) (41).

From the total 1670 participants, 1648 and 1647 samples were analyzed for serum folate and RBC folate concentration, respectively. The descriptive statistical analysis includes median, IQR, and prevalence of RBC and serum folate deficiency. The analysis was performed using R software, version 4.1.1 (R Core Team).

#### Farming system data

The farming system data used in the present study were obtained from the Famine Early Warning System Network (FEWS NET; https://fews.net) and IFPRI Harvest Choice (https://harvestchoice.org/products/data) databases. The methodology and description of the farming system data set have been reported elsewhere (31).

#### Geospatial data analysis

Serum and RBC folate concentrations were mapped at the national level for WRA. Before the spatial analysis of these data, summary statistics and plots were examined to check the plausibility of the assumption that the data are realizations of normally distributed random variables (**Supplemental Information 1**). This is not a strict assumption of ordinary kriging, but it improves the efficiency of statistical estimators, and facilitates the comparison of spatial models by cross-validation (42). On the basis of the summary statistics it was decided to transform both variables to natural logarithms. The predictive maps for serum and RBC folate concentrations were made by obtaining the ordinary kriging on nodes of a 60-m square grid which can then be visualized in a geographic information system (GIS) environment as a continuous surface (see **Supplemental Information 2**).

### Ethical approval

The ENMS was approved by the National Research Ethics Review Committee at the Ministry of Science and Technology, Ethiopia (Reference 3.10/433/06). Written informed consent was obtained from the parti-cipants. This study was also approved by the Research Ethical Review Committee at the Ethiopian Public Health Institute (Protocol EPHI-IRB-140–2018) and Institutional Review Board at Addis Ababa University (Reference CNCSDO/192/14/21).

## Results

### Characteristics of study participants

**Table 1** presents the demographic characteristics of the participants in the present study. The majority of the participants were living in rural residences, with low levels of literacy and from the lowest wealth category.

**TABLE 1:** Characteristics of study participants and serum and RBC folate concentrations among nonpregnant women of reproductive age in the ENMS^1^

Characteristics	*n*	Serum folate,^2^ nmol/L	RBC folate,^2^ nmol/L
Age, y			
15–19	313	12.0 [8.6–18.2]	535.8 [415.5–790.9]
20–29	618	11.9 [8.0–19.6]	548.7 [347.0–891.0]
30–39	482	13.4 [8.8–21.5]	592.8 [415.3–815.8]
40–49	233	13.1 [9.3–22.4]	626.1 [415.9–917.9]
Residence			
Urban	588	11.5 [8.7–18.1]	563.2 [426.7–856.4]
Rural	1058	12.4 [8.4–21.0]	567.3 [389.0–844.9]
Educational status			
Illiterate	758	13.6 [8.9–21.3]	592.8 [394.3–894.0]
Primary	505	11.3 [8.3–20.4]	529.6 [394.8–790.9]
Secondary	281	10.8 [7.3–16.7]	526.8 [382.7–813.2]
Higher	112	12.4 [9.8–16.2]	624.0 [458.4–860.1]
Wealth tertiles			
Lowest	1047	12.3 [8.5–21.0]	573.9 [396.3–855.9]
Middle	417	11.5 [8.3–17.3]	493.9 [373.5–799.1]
Highest	181	12.6 [8.7–19.6]	597.7 [418.9–800.7]

1 Values are median [IQR] unless otherwise indicated. ENMS, Ethiopian National Micronutrient Survey.

2 The estimation was weighted using the ENMS sampling weight factor.

### Prevalence of folate deficiency

The median [IQR] serum folate and RBC folate concentrations were 12.3 nmol/L [8.5–20.3 nmol/L] and 567.3 nmol/L [394.9–846.6 nmol/L], respectively. Both serum and RBC folate concentrations varied by region (**Table 2**). The national prevalence of folate deficiency among WRA based on serum and RBC folate concentrations, using homocysteine concentrations as the metabolic indicator, was 11.6% and 5.7%, respectively. The majority (77.9%) of the women had suboptimal folate status indicating increased risk of NTD-affected pregnancies, with significant variation among regions ranging from 100.0% in Afar to 60.9% in Southern Nations, Nationalities, and People's Region (SNNPR) (**Table 3**).

**TABLE 2:** Serum and RBC folate concentrations among nonpregnant women of reproductive age by region of residence in the ENMS^1^

Regions	*n*	Serum folate,^2^ nmol/L	RBC folate,^2^ nmol/L
Addis Ababa	174	13.3 [9.5–19.6]	512.0 [395.6–773.6]
Afar	109	8.7 [7.1–11.2]	408.8 [304.7–583.0]
Amhara	233	12.0 [8.5–17.9]	615.4 [450.2–924.3]
Benishangul-Gumuz	103	11.5 [7.3–15.8]	458.6 [309.1–657.9]
Dire Dawa	97	11.8 [8.4–17.4]	486.6 [358.8–718.1]
Gambella	116	11.9 [8.1–18.4]	521.5 [371.6–745.4]
Harari	91	9.8 [7.3–12.1]	383.1 [313.3–536.0]
Oromia	246	12.3 [8.7–20.7]	516.4 [385.1–719.4]
SNNPR	195	15.3 [9.2–30.2]	721.5 [418.9–1177.9]
Somali	101	7.4 [5.4–11.0]	459.1 [324.6–691.5]
Tigray	182	11.8 [7.5–17.7]	476.0 [344.5–723.7]
National	1647	12.3 [8.5–20.3]	567.3 [394.9–846.6]

1 Values are median [IQR] unless otherwise indicated. ENMS, Ethiopian National Micronutrient Survey; SNNPR, Southern Nations, Nationalities, and People's Region.

2 The estimation was weighted using the ENMS sampling weight factor.

**TABLE 3:** Prevalence of folate deficiency by study characteristics among nonpregnant women of reproductive age of the ENMS^1^

	Prevalence of folate deficiency^2^		Prevalence of suboptimal folate status indicating increased risk of neural tube defect–affected pregnancies^2^
Variables	Serum folate	RBC folate	
Regions			
Addis Ababa	7.0 (12)	3.8 (6)	82.4 (147)
Afar	22.6 (25)	9.6 (9)	100.0 (109)
Amhara	11.3 (26)	2.1 (6)	72.9 (169)
Benishangul-Gumuz	16.8 (17)	14.1 (15)	95.2 (98)
Dire Dawa	9.5 (8)	4.4 (4)	89.2 (88)
Gambella	16.8 (19)	4.3 (4)	80.2 (92)
Harari	19.9 (17)	9.6 (9)	96.9 (88)
Oromia	10.0 (24)	8.0 (22)	87.0 (214)
SNNPR	9.5 (20)	5.0 (11)	60.9 (121)
Somali	38.6 (38)	15.2 (11)	85.8 (89)
Tigray	14.0 (28)	3.7 (8)	86.5 (159)
National	11.6 (234)	5.7 (105)	77.9 (1374)
Age, y			
15–19	10.8 (37)	5.6 (17)	80.8 (262)
20–29	15.3 (106)	6.4 (44)	75.7 (519)
30–39	9.8 (63)	4.0 (29)	80.9 (408)
40–49	6.3 (28)	7.6 (15)	73.4 (185)
Residence			
Urban	7.7 (64)	3.2 (30)	78.2 (505)
Rural	12.6 (170)	6.4 (75)	77.9 (869)
Educational level			
Illiterate	9.9 (117)	6.0 (54)	75.4 (622)
Primary	12.6 (68)	5.5 (31)	81.3 (423)
Secondary	8.5 (39)	0.9 (17)	77.1 (238)
Higher	10.7 (10)	6.7 (3)	80.3 (91)
Wealth tertiles			
Lowest	11.8 (162)	6.2 (76)	76.8 (848)
Middle	12.1 (57)	6.8 (25)	82.5 (364)
Highest	7.2 (15)	3.4 (4)	83.5 (162)

1 Values are % (*n*). ENMS, Ethiopian National Micronutrient Survey; SNNPR, Southern Nations, Nationalities, and People's Region.

2 The estimation of the percentage of prevalence was weighted using the ENMS sampling weight factor but the frequencies are from unweighted samples.

### Spatial variation of serum and RBC folate concentrations

The spatial variation of serum and RBC folate concentrations was explored for WRA. The variograms for RBC folate and serum folate (**Supplemental Figure 1**) showed spatial dependence at distances of ≤ 300 km and ≤ 200 km, respectively.

The cross-validation errors for the model fitted to the estimates using Matheron (43) appeared to be normally distributed (**Supplemental Figure 2**), with a median standardized squared prediction error (SSPE) of 0.39 for serum folate and 0.33 for RBC folate. The values of SSPE fell within the (95% CI: 0.33, 0.58) expected value, and so we used the variogram models based on Matheron's estimator for both variables. RBC folate had a nugget variance of 0.099, a correlated variance of 0.069, and a distance parameter of 93.4 km. For serum folate nugget variance was 0.123, correlated variance was 0.113, and the distance parameter was 67.5 km. For both variables the nugget variance—which describes the variation at finer scales than can be resolved by the sampling scheme (and analytical error)—was of a similar order to the correlated variance, indicating that the fine-scale variation was important. The spatially correlated variance showed dependence up to a distance of ∼3 times the distance parameter.

**FIGURE 2:**
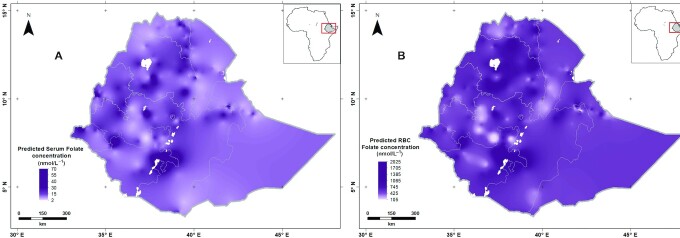
Predicted folate concentration (the mean of the prediction distribution) based on serum folate (A) and RBC folate (B) in women of reproductive age in Ethiopia. Created using ArcGIS 10.4.1. ESRI ArcGIS Desktop: Release 10. Environmental Systems Research Institute, Redlands (2011).

**Figure 2** shows the predicted serum and RBC folate concentrations for WRA across Ethiopia obtained by ordinary kriging. High concentrations of serum folate were observed in the northwest, west, south, and southeast including Amhara, Benshangul-Gumuz, Gambella, Addis Ababa, and southwest Oromia, whereas low serum folate concentrations were observed in northern, northeastern, and southern parts including Tigray, Afar, and southern Oromia.

**FIGURE 3:**
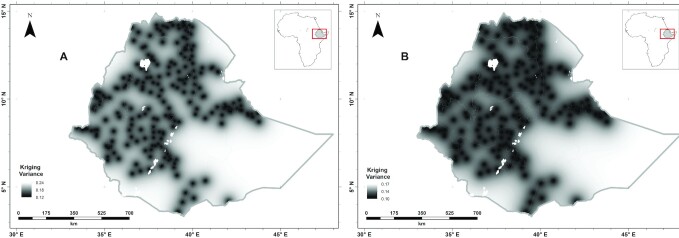
Folate concentration based on serum folate (A) and RBC folate (B) kriging variance (the variance of the prediction distribution) in women of reproductive age in Ethiopia. Created using ArcGIS 10.4.1. ESRI ArcGIS Desktop: Release 10. Environmental Systems Research Institute, Redlands (2011).

As described previously for Se (25) and Zn (24), the interpolation errors are plotted in **Figure 3**; the highest prediction error is observed in light gray colors in both panels. Figure 3 shows the kriging variances of the predictions of serum and RBC folate concentrations for WRA; the prediction kriging variance or prediction error is expected to visualize the uncertainty of the prediction. As can be observed in Figure 3, in some areas of Ethiopia, especially in the east, northeast, south, and southeast, the kriging variance was high owing to sparse observations; in this situation, additional sampling is essential for appropriate intervention.

The larger RBC folate concentrations were found in the northwest, west, southwest, and southeast of Ethiopia, whereas smaller RBC folate concentrations were observed in the north, south, southeast, and southwest parts of the country. Figure 3 shows the kriging variances of the predictions of RBC folate concentration for WRA; the uncertainty of the prediction was high in the areas where the observations were sparse.

**FIGURE 4:**
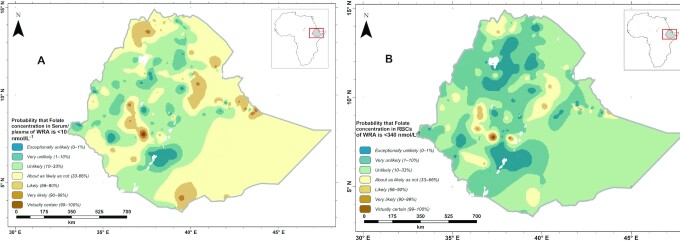
Probability that folate concentrations of WRA in the Ethiopia National Micronutrient Survey fall below the nutritionally significant threshold [serum folate < 10 nmol/L (A); RBC folate < 340 nmol/L (B)]. Created using ArcGIS 10.4.1. ESRI ArcGIS Desktop: Release 10. Environmental Systems Research Institute, Redlands (2011). WRA, women of reproductive age.

Compared to general measures of uncertainty such as prediction intervals, probability maps based on nutritionally significant thresholds are easily understandable and preferred by stakeholders for use of spatial information (44). **Figure 4** shows a map of the probability that folate concentration falls below the cutoff for adequacy: serum folate < 10 nmol/L (Figure 4A) and RBC folate < 340 nmol/L (Figure 4B) using homocysteine concentrations as the metabolic indicator. We used “calibrated phases” of the Intergovernmental Panel for Climate Change (45) in the legend to support readers to interpret the uncertainty in estimating the probability that folate concentrations are below or above a nutritionally significant threshold. The probability map shows that women in large parts of the country including the northwest, central, southwest, and eastern parts of Tigray are very unlikely to exceptionally unlikely to have a low RBC folate concentration. Also, WRA in the east, southeast, southwest, and western parts of Tigray are likely through virtually certain to have a low serum concentration.

### The association between farming system and folate deficiency

**FIGURE 5:**
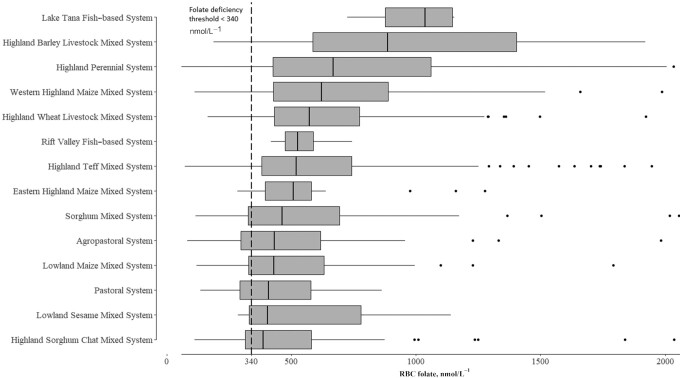
RBC folate concentration of nonpregnant women of reproductive age by farming system in Ethiopia. The vertical dashed line represents the RBC folate concentration threshold for deficiency. The boxes contain the median, and lower and upper quartile of the data set; whiskers indicate the variability outside the upper or lower quartiles; individual dots are outliers.

There was considerable variability of RBC folate concentration among farming systems (**Figure 5**). A high concentration of RBC folate was observed among populations from the Lake Tana fish-based system (median: 1036 nmol/L) and highland barley livestock mixed farming systems (median: 886.0 nmol/L). On the other hand, low concentrations were observed among populations from the pastoral (median: 408.8 nmol/L), lowland sesame mixed (median: 403.8 nmol/L), and highland sorghum chat mixed farming systems (median: 386.7 nmol/L).

## Discussion

This new analysis reports evidence of the geospatial structure of folate status among adult women in Ethiopia and estimates the association of adult women's folate status with local farming systems. Approximately 1 in 10 WRA had low serum folate concentrations and ∼1 in 20 had low RBC folate concentrations. This estimated prevalence of deficiency is lower than that reported in the ENMS, i.e., 17.3% and 32% of women with low serum and RBC folate concentrations, respectively. This is because, unlike the ENMS, this study did not adjust serum and RBC folate concentrations for inflammation which is consistent with recommendations from the Biomarkers Reflecting Inflammation and Nutritional Determinants of Anemia (BRINDA) consortium. BRINDA is a global collaboration to improve micronutrient assessment and anemia (46).

Maternal folate deficiency has been linked to increased risk of NTD-affected pregnancies. About 78% of WRA in the present study had a low RBC folate concentration indicative of an increasing risk of giving birth to infants with NTDs. Iron–folic acid (IFA) supplementation specifically to adolescent girls and WRA and wheat flour fortification to the general public are part of nutrition strategies in Ethiopia to address folate deficiency. However, a report shows that only pregnant women mainly during antenatal care (ANC) visits consume IFA tablets and 77% of women in Ethiopia missed ≥ 1 ANC visit, and of those women with ≥ 1 visit, 69% did not obtain IFA. Of the women who obtained IFA, 4% did not consume any tablets and almost all (99%) pregnant women did not consume the minimum required number of tablets (47). Starting time of and adherence to IFA supplementation are further limitations to curbing folate deficiency (48). Voluntary fortification of wheat flour with multiple mineral and vitamin micronutrients including folate is legislated; however, it is unlikely to benefit populations in areas where wheat consumption is low, or where wheat is processed at small-scale milling houses which do not have fortification facilities.

Folate status among WRA in Ethiopia is highly spatially variable. The geostatistical analysis revealed that folate status is spatially dependent at distances ≤ 300 km. High concentrations of serum folate were found in parts of western, central, and southern Ethiopia whereas low concentrations were observed among women from northern and eastern parts of the country. Several factors may contribute to the variation in folate concentration, including the food system, which is highly localized in Ethiopia, where foods are sourced mainly through subsistence and small-scale production or purchase of locally produced foods (49). Green leafy vegetables, legumes, and fruits are the main dietary sources of folate (50) and production and consumption of these food items are mostly localized and determined by the farming system. However, consumption of fruits and vegetables among women in Ethiopia is extremely low (51). Unfortunately, spatial information on production of food items in Ethiopia is not available. Fortified cereals such as wheat flour could greatly increase dietary folate intakes; however, the flour fortification program in the country is only at the preparatory phase. The majority of women in the present study had a low literacy rate which may contribute to the deficiency. Literacy and schooling of women are important to enhance their nutrition knowledge and expand opportunities to improve allocation of resources to access diversified and nutrient-dense foods (52).

Ethiopia has a highly differentiated agroecology and is characterized by diversified farming systems which directly or indirectly play important roles in food and nutritional security. A study identified and reported the presence of 16 farming systems in Ethiopia (31). In the present study, women from the Lake Tana fish-based system (which covers most of the south Gonder and west Gojjam zones of Amhara) (Figure 1) and highland barley livestock mixed system had the greatest folate concentrations, whereas the lowest folate concentrations were observed among women from the highland sorghum chat and mixed chat systems (northwestern to eastern Hararghe), lowland sesame mixed system, and pastoral systems. The pastoral system is characterized by keeping livestock including cattle, camels, small ruminants, and equines (31). However, despite possession of large numbers of livestock, consumption of animal source foods except milk is low (53). Cow milk or goat milk is known to contain low amounts of folate (54).

In the current study, we observed a high prevalence of suboptimal folate status indicating increased risk of NTD-affected pregnancies, which is consistent with dietary data showing low consumption of legumes, fruits, and green leafy vegetables in Ethiopia. The Ethiopian National Food Consumption Survey (NFCS) also reported that women in the Afar and Somali regions consumed greater quantities of milk than in other regions (55), which is consistent with the observed high prevalence of folate deficiency and risk of NTD-affected pregnancies in the Afar and Somali regions. Animal milk contains low concentrations of folate (54). In addition, compared with women from other regions, those from SNNPR and Gambella had greater consumption of fruits and vegetables (55). In the present study, women from SNNPR had a lower prevalence of folate deficiency than those from other regions. Folate status was variable within regions, and agro-ecological zone may be a better predictor of folate status. Analysis of the Ethiopian NFCS data by agro-ecological zone is warranted but is precluded by a lack of geographic data.

The Lake Tana fish-based farming system is dominated by maize, rice, teff, pulses, fish, and livestock production. It is also known for production of vegetables such as onion and tomato. Barley and potato are the 2 dominant crops followed by oats and pulses including faba bean for the highland barley livestock mixed system. Sheep are the dominant livestock type, with a small number of cattle for milk production. On the other hand, the highland sorghum chat mixed system is known for sorghum and the commercial crop, chat. In addition, it is also known for production of sweet potato, beans, and maize (31). Organ meats such as liver, green leafy vegetables, pulses, and whole grains are all good sources of folate. In addition, teff flour is an important dietary source of folate (mean ± SD: 59 ± 11 μg/100 g dry matter), which is comparable with cereals more widely known for their high folate content such as oats. However, significant amounts of folate could be lost during thermal processing to make injera (a partially fermented flatbread, traditional in Ethiopia), and this warrants further investigation given the importance of teff in many Ethiopian diets. The average (mean ± SD) folate content of teff-injera is 39 ± 8 μg /100g dry matter (56).

The results in the present study can be used as baseline information for the targeting and design of future studies, including studies to test the efficacy and effectiveness of interventions and programs aiming to alleviate folate deficiency. In addition, the geostatistical information including kriging variance results may inform future folate surveillance work. The strengths of this study include its large sample size, use of serum and RBC folate concentrations to catch acute and chronic folate concentrations of WRA, and the use of geostatistical modeling to predict folate concentrations at unsampled locations, and associated estimates of the uncertainty. On the other hand, only a small number of samples or no samples were collected during the ENMS in parts of Somali, Afar, and southern Oromia owing to inaccessibility, and information on population folate status in these regions is therefore very limited.

In conclusion, the majority of women (78%) in Ethiopia had a low folate concentration, increasing the risk of NTD-affected pregnancies. Higher folate concentrations were found among women from western, central, and parts of southern Ethiopia, whereas lower folate concentrations were observed in northern and eastern parts of the country. Folate deficiency was associated with farming systems. Women from the Lake Tana fish-based system had higher folate status, whereas those from the highland sorghum chat mixed system, lowland sesame mixed system, and pastoral and agropastoral systems had relatively lower folate status. The results of the present study suggest the pressing need to develop food fortification interventions, dietary diversification, and folic acid supplementation to alleviate folate deficiency and improve maternal and child health in affected areas. In addition, agronomic biofortification to enhance folate content of staple crops by plant breeding, which is a promising cost-effective strategy, could complement the aforementioned interventions to control folate deficiency. Folate biofortification intervention is especially important to the economically disadvantaged segment of the population in remote areas where other nutrition strategies are less accessible (57).

## Data Availability

Owing to the agreement with the Ethiopian Public Health Institute (EPHI), data described in the article will not be made available. Requests for data access should be directed to the EPHI.

## Notes

Supported in part by Bill & Melinda Gates Foundation grant INV-002855 through the Micronutrient Action Policy Support (MAPS) project. Under the grant conditions of the Foundation, a Creative Commons Attribution 4.0 Generic License has already been assigned to the Author Accepted Manuscript version that might arise from this submission. The funder had no role in the design, execution, analyses, or interpretation of the data.

Author disclosures: The authors report no conflicts of interest.

Supplemental Figures 1 and 2 and Supplemental Information 1 and 2 are available from the “Supplementary data” link in the online posting of the article and from the same link in the online table of contents at https://academic.oup.com/cdn/.

BGS and HT contributed equally to this work.

Abbreviations used: ANC, antenatal care; BRINDA, Biomarkers Reflecting Inflammation and Nutritional Determinants of Anemia; ENMS, Ethiopia National Micronutrient Survey; IFA, iron–folic acid; LOD, limit of detection; LOQ, limit of quantification; NFCS, National Food Consumption Survey; NTD, neural tube defect; QC, quality control; SNNPR, Southern Nations, Nationalities, and People's Region; SSPE, Standardized Squared Prediction Error; WRA, women of reproductive age.
